# Calcium phosphate-hybridized tendon graft to enhance tendon-bone healing two years after ACL reconstruction in goats

**DOI:** 10.1186/1758-2555-3-31

**Published:** 2011-12-14

**Authors:** Hirotaka Mutsuzaki, Masataka Sakane

**Affiliations:** 1 Department of Orthopaedic Surgery, Ibaraki Prefectural University of Health Sciences, 4669-2 Ami Ami-machi, Inashiki-gun, Ibaraki 300-0394, Japan; 2 Department of Orthopaedic Surgery, Institute of Clinical Medicine, Graduate School of Comprehensive Human Sciences, University of Tsukuba, 1-1-1 Tennodai, Tsukuba, Ibaraki 305-8575, Japan

**Keywords:** ACL reconstruction, tendon-bone healing, calcium phosphate hybridization, direct insertion, bone tunnel enlargement

## Abstract

**Background:**

We developed a novel technique to improve tendon-bone attachment by hybridizing calcium phosphate (CaP) with a tendon graft using an alternate soaking process. However, the long-term result with regard to the interface between the tendon graft and the bone is unclear.

**Methods:**

We analyzed bone tunnel enlargement by computed tomography and histological observation of the interface and the tendon graft with and without the CaP hybridization 2 years after anterior cruciate ligament (ACL) reconstruction in goats using EndoButton and the postscrew technique (CaP, n = 4; control, n = 4).

**Results:**

The tibial bone tunnel enlargement rates in the CaP group were lower than those in the control group (*p *< 0.05). In the CaP group, in the femoral and tibial bone tunnels at the anterior and posterior of the joint aperture site, direct insertion-like formation that contained a cartilage layer without tidemarks was more observed at the tendon-bone interface than in the control group (*p *< 0.05). Moreover, the gap area between the tendon graft and the bone was more observed at the femoral bone tunnel of the joint aperture site in the control group than in the CaP group (*p *< 0.05). The maturation of the tendon grafts determined using the ligament tissue maturation index was similar in both groups.

**Conclusions:**

The CaP-hybridized tendon graft enhanced the tendon-bone healing 2 years after ACL reconstruction in goats. The use of CaP-hybridized tendon grafts can reduce the bone tunnel enlargement and gap area associated with the direct insertion-like formation in the interface near the joint.

## Introduction

The anterior cruciate ligament (ACL) is the most frequently injured ligament in the knee. Surgical reconstruction using a replacement graft is the preferred method of treatment. A semitendinosus-gracilis (STG) tendon graft, the so-called soft tissue graft, is commonly used [1,2]. However, the STG tendon graft requires soft-tissue-to-bone healing within both bone tunnels. Grana et al. [3] observed indirect bonding with fibrous tissue between a hamstring tendon autograft and bone tunnels in a rabbit ACL reconstruction model. The indirect bonding formation at the interface is similar to that observed after a long period [4]. Many studies attempted to improve the healing of tendon to bone with different therapeutic modalities including application of periosteum augmentation, bone morphogenetic protein, calcium-phosphate cement, granulocyte colony-stimulating factor, gene transfer, and so on [5-11]. We developed a novel technique to improve tendon-bone attachment by hybridizing calcium phosphate (CaP) with tendons using an alternate soaking process [12]. Using the CaP-hybridized tendon, we observed a scarless direct bonding area between the tendon graft and the bone without inflammation two to three weeks after ACL reconstruction in rabbits [13,14], which was also observed in goats [15]. The CaP-hybridized tendon graft reduced bone tunnel enlargement in the femoral side 6 months after ACL reconstruction in goats [16]. Moreover, the anterior-posterior translations in the reconstructed knees in the CaP group were shorter and the corresponding in situ forces were greater than those in the control group at full extension and 60° of knee flexion 1 year after the ACL reconstruction in goats [17]. The new bone formation at the bone tunnel and cartilage layer formation at the tendon-bone interface near the joint in the CaP group were more observed than those in the control group [17]. Commonly, a minimum of two years of follow-up after ACL reconstruction is recommended in order to evaluate the clinical results. However, the long-term effect of the CaP-hybridized tendon after ACL reconstruction in animal experiments is unclear. To clarify this issue, we used a goat model of ACL reconstruction, because long-term studies of goat knees have shown the effective restoration of knee stability after ACL reconstruction [18,19].

We considered that an appropriate mechanical stress at the interface after bonding between the tendon graft and the bone, and a prerequisite firm anchoring may promote direct insertion-like formation at the tendon-bone interface and the maturation of the tendon graft [13-17]. Therefore, we hypothesized that cartilaginous anchoring formation between the tendon graft and the bone is more mature, and both the bone tunnel enlargement and the percentage of the gap area at the interface that is loosening 2 years after ACL reconstruction are smaller when using the CaP hybridization method than when using the conventional method, because the anchoring formation in the CaP group (direct bonding and cartilaginous anchoring) is different from that in the control group (fibrous bonding) from 2 weeks to 1 year after the operation [13-17]. Moreover, the graft in the CaP group may be more mature than that in the control group, because of its anchoring formation associated with an appropriate mechanical stress.

The objective of this study was to analyze bone tunnel enlargement by computed tomography (CT) and histological observation of the interface and the tendon graft in the group with the CaP-hybridized tendon graft and in the group with untreated tendon graft 2 years after ACL reconstruction using a goat model.

## Materials and methods

### CaP hybridization method

Eight skeletally mature female Saanen breed goats (50-70 kg) were used in this study. The goats were maintained in accordance with the guidelines of the Ethical Committee of the Biomaterial Center of the National Institute for Materials Science and the National Institutes of Health guidelines for the care and use of laboratory animals (NIH Pub. No. 85-23 Rev. 1985).

**Figure 1:**
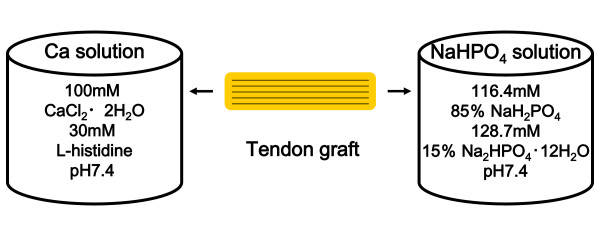
**Schema of CaP hybridization method**. Flexor digitorum longus (FDL) tendon is soaked in a Ca solution (100 mM CaCl_2 _+ 30 mM L-histidine, pH 7.4, 280 mOsm/l). The grafts are subsequently soaked in a NaHPO_4 _solution (116.4 mM NaH_2_PO_4_:128.7 mM Na_2_HPO_4 _12H_2_O = 15%:85%, pH 7.4, 280 mOsm/l). The temperatures of the solution and room are both 25°C. Before each soaking, the grafts are washed in a saline solution. This cycle is repeated ten times.

Flexor digitorum longus (FDL) tendons were used in this study. Double-strand FDL tendons of 45 mm length and 5.5 mm diameter were prepared. The tibial end of the grafts was secured using the Krackow technique with No. 2 nonabsorbable sutures (ETHIBOND* EXCEL, ETHICON, INC., USA), and a polyester tape suture tied over the EndoButton (Smith & Nephew, Andover, MA, USA) was passed through the looped femoral end of the grafts. Then, the central third of the grafts, considered as the intra-articular portion, was covered with the sleeve of a rubber glove tied on each side with the No. 2 nonabsorbable sutures to prevent CaP hybridization [15-17]. After these procedures, the grafts were soaked in 100 ml of a Ca solution (100 mM CaCl_2 _+ 30 mM L-histidine, pH 7.4, 280 mOsm/l). The grafts were subsequently soaked in 100 ml of a NaHPO_4 _solution (116.4 mM NaH_2_PO_4_:128.7 mM Na_2_HPO_4 _12H_2_O = 15%:85%, pH 7.4, 280 mOsm/l) (Figure 1). The temperatures of the solution and room were both 25°C. Before each soaking, the grafts were washed in a saline solution. This cycle was repeated ten times [13-17]. As the control, the tendon was soaked in a saline solution for 10 minutes.

### Surgical procedures

All the surgical procedures were performed under sterile conditions with the animals under general anesthesia. On the right knee, an anterior lateral skin incision was made. The ACL was then completely transected and the gross anterior subluxation of the tibia was confirmed by manual examination. We drilled using a Kirschner wire from the anteromedial surface of the proximal tibia to the tibial insertion of the ACL. Then we drilled femoral side straightly with the knee positioned in 45° flexion to ensure consistency of surgical technique. The bone tunnel was reamed using a 5.5-mm-diameter canulated drill, resulting in the bone tunnel in the femoral side opening anterior of the femoral insertion. The length of the tunnel was at least 20 mm.

The graft described above was passed through the femoral and tibial tunnels and fixed to the anteromedial surface of the tibia with 20 N as the initial tension using a 4.5-mm-diameter cortex screw (MEIRA Corporation, Nagoya, Japan) [15-17].

**Figure 2:**
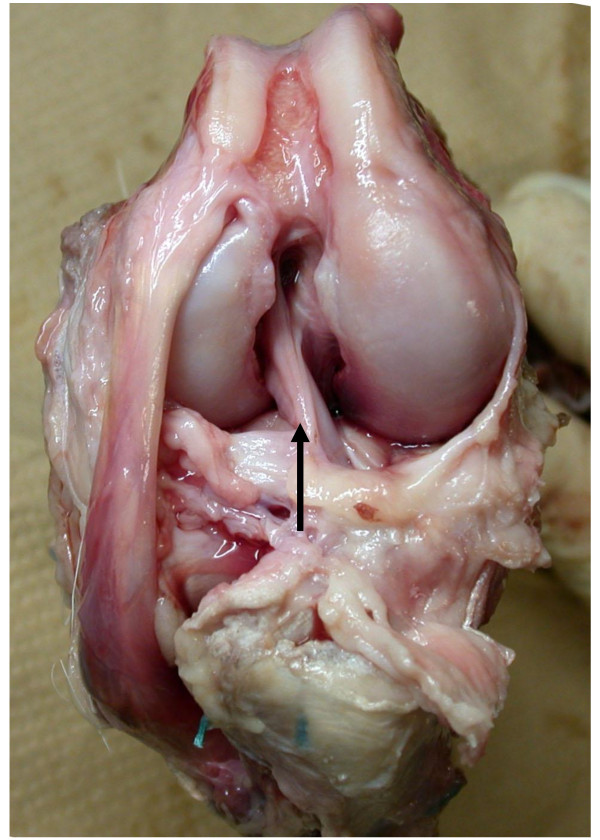
**Two years after ACL reconstruction**. The ACL replacement graft remained (arrow).

Postoperatively, all the goats were allowed free cage activity (cage area, 50 m^2^). All the goats tolerated the operation well and were partially weightbearing within a few hours after surgery. However, visual inspection revealed that normal gait patterns did not return until 3 to 4 weeks after the surgery. All ACL replacement grafts remained 2 years after the operations (Figure 2).

### Ex vivo CT

The four frozen specimens of goats from each group were used for CT (Brilliance CT 64, Philips, Amsterdam, Netherlands) to assess femoral and tibial bone tunnels. The CT (voltage: 120 kV, current: 230 mA) in the full extension knee position was supervised and the obtained CT images were analyzed by a single radiologist. A standard protocol was used consistently throughout the study. Initial volume acquisition was with 0.9 mm slices from 30 mm above the femoral tunnel to 30 mm below the tibial tunnel. Using the work station of Virtual Place Lexus (AZE Ltd., Tokyo, Japan), three-dimensional images were reconstructed. Axial images of the femoral and tibial bone tunnels were obtained using the reconstructed three-dimensional images. We measured the tunnel cross-sectional area (CSA) of the femur and tibia at the main joint aperture site using the axial images of the femoral and tibial bone tunnels. The rate of increase in tunnel CSA was calculated using the following formula: CSA increase rate (%) = (CSA at 2 years - Initial CSA) × 100/Initial CSA [16]. Initial CSA is calculated on the basis of the diameter of the reamer.

### Histological analysis

**Figure 3:**
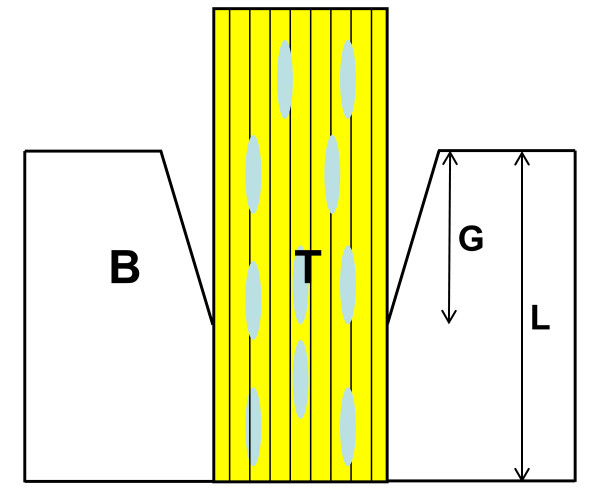
**Schematic view of a gap area between a tendon graft and bone**. T = tendon graft, B = bone, G = length of a gap area from a joint aperture site, L = length of a tendon graft in a bone tunnel. Gap area percentage (%) = (G - L) × 100/L.

After the CT analysis, the femur-ACL graft and ACL graft-tibia complex were harvested. At each period, four specimens from each group were fixed in 10% neutral buffered formalin, decalcified, and embedded in paraffin. The specimens were sliced 5 μm thick parallel to the long axis of the bone tunnel and then stained with hematoxylin and eosin (H-E) and safranin-O to identify the cartilage layer in the interface. The specimens were examined by light microscopy after staining (BX-51, Olympus Optical Co., Ltd., Tokyo, Japan). The tendon-bone interface was histologically compared between the CaP and control groups. The interface between the tendon and the bone tunnel was assessed by observing its formation (fibrous bonding, cartilaginous insertion and gap area at joint aperture site) at both the anterior and posterior bone tunnels and on both femoral and tibial sides. Moreover, the percentage of the gap area in the bone tunnel was calculated using the following formula: Gap area percentage (%) = (length of the gap area from the joint aperture site - length of the tendon graft in the bone tunnel) × 100/length of the tendon graft in the bone tunnel (Figure 3).

The ligament tissue maturation index (LTMI) of Murray et al. [20] was used to evaluate the maturation of tendon grafts according to the following 3 criteria: [1] cellular aspects including cell density, nuclear shape, and orientation; [2] extracellular matrix characteristics, such as crimp; and [3] vascular features including blood vessel density and maturity (total score, 28 points).

### Statistical Analyses

The bone tunnel enlargement data and histological analysis of the two groups were compared using Student's *t-*test at a *p *< 0.05 significance value. To compare the histological difference between the CaP and control groups, Mann-Whitney's U test was used at a *p *< 0.05 significance value.

## Results

### Ex vivo CT

**Figure 4:**
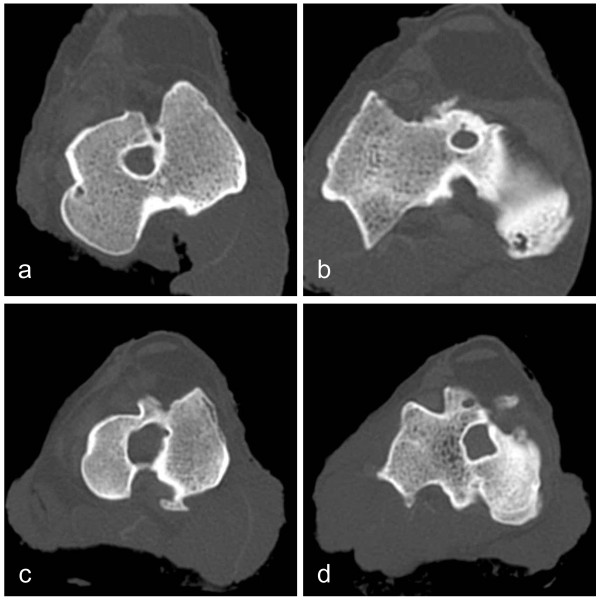
**CT images of cross-sectional area of tunnel**. (a) Femoral bone tunnel in CaP group. (b) Tibial bone tunnel in CaP group. (c) Femoral bone tunnel in control group. (d) Tibial bone tunnel in control group. The femoral and tibial bone tunnels in the control group showed greater enlargement than those in the CaP group.

Bone tunnel enlargement in the control group was greater than that in the CaP group. The bone tunnel enlargement rate in the tibial bone tunnel in the CaP group was significantly smaller than that in the control group. The bone tunnel enlargement rate in the femoral bone tunnel CSA with the untreated tendon graft was 143.3 ± 116.8% and that with the CaP-hybridized tendon graft was 122.1 ± 77.9 (*p *= 0.3861). That in the tibial bone tunnel CSA with the untreated tendon graft was 215.9 ± 106.2% and that with the CaP-hybridized tendon graft was 87.9 ± 64.8 (*p *= 0.0427) (Figure 4).

### Histological Findings

**Figure 5:**
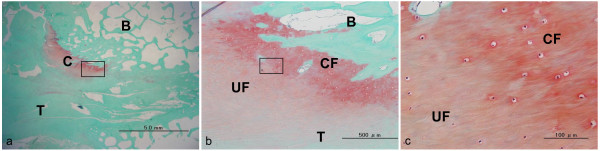
**Histological sections stained with safranin-O for the CaP group**. This area is the posterior of the aperture site in the femur. T = tendon graft, B = bone, C = cartilage tissue, UF = uncalcified fibrocartilage-like tissue, CF = calcified fibrocartilage-like tissue. (a) Low-magnification image (x 12.5). The cartilage layer with glycosaminoglycan stained red was observed between the tendon graft and the bone. (b) Magnified views of boxed part in (a) (x 100). The interface shows 4 distinct layers, namely, tendon graft, uncalcified fibrocartilage, calcified fibrocartilage layer, and bone tunnel. (c) Magnified views of the boxed part in (b) (x 400). The cartilage layer shows 2 distinct layers, namely, uncalcified fibrocartilage and calcified fibrocartilage layer without tidemarks.

**Figure 6:**
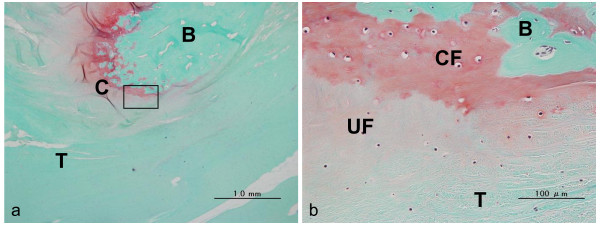
**Histological sections stained with safranin-O for CaP group**. This area is the anterior of the aperture site in the tibia. T = tendon graft, B = bone, C = cartilage tissue, UF = uncalcified fibrocartilage-like tissue, CF = calcified fibrocartilage-like tissue. (a) Low-magnification image (x 40). The cartilage layer with glycosaminoglycan stained red was observed between the tendon graft and the bone. (b) Magnified views of the boxed part in (a) (x 400). The cartilage layer shows 2 distinct layers, namely, uncalcified fibrocartilage and calcified fibrocartilage layer without tidemarks.

The results of histological analysis (CaP, n = 4; control, n = 4) are shown in Tables 1, 2, and 3. The formation of a cartilage layer in the interface in the CaP group was more observed than in the control group (*p *= 0.0352). In the femoral and tibial bone tunnels at the anterior and posterior of the aperture site, the cartilage layers were on average 150 μm to 2.6 mm in length and 80 μm to 550 μm in thickness, and the staining of glycosaminoglycan by safranin-O was observed. In each site, the length and the thickness were not significant difference between the CaP and control groups (*p *> 0.05). The cartilage layer showed two distinct layers, namely, uncalcified fibrocartilage and calcified fibrocartilage layers without tidemarks (Figures 5, 6). At the aperture site, cartilage layers between the tendon and the bone were observed at the anterior surface of the tibial bone tunnel in all 4 specimens, at the posterior surface of the tibial bone tunnel in 3 specimens, at the anterior surfaces of the femoral bone tunnel in 2 specimens, and at the posterior surface of the femoral bone tunnel in 2 specimens. In the tibial bone tunnel, the cartilage layer was more prominent than that in the femoral bone tunnel.

**Table 1:** Histological observation (cartilage layer formation)

	Femur (N = 4)		Tibia (N = 4)	
	**anterior**	**posterior**	**anterior**	**posterior**

CaP group (n = 4)	2/4	2/4	4/4	3/4
Control (n = 4)	0/4	1/4	3/4	1/4

**Table 2:** Histological observation (red stained area by Safranin-O staining in cartilage layer at interface)

	Femoral side				Tibial side			
	**anterior**		**posterior**		**anterior**		**posterior**	

	**length (mm)**	**width (mm)**	**length (mm)**	**width (mm)**	**length (mm)**	**width (mm)**	**length (mm)**	**width (mm)**

CaP group (n = 4)	0.15 ± 0.29	0.08 ± 0.16	2.65 ± 3.15	0.56 ± 0.68	1.45 ± 0.76	0.35 ± 0.16	1.15 ± 1.38	0.22 ± 0.18
Control (n = 4)	0	0	0.42 ± 0.85	0.09 ± 0.17	0.56 ± 0.54	0.21 ± 0.24	0.28 ± 0.55	0.08 ± 0.16

Results are the mean ± SD

**Table 3:** Histological observation (gap area at interface)

	Femur		Tibia	
	**N = 4**		**N = 4**	

	**gap area rate: AV ± SD %**		**gap area rate: AV ± SD %**	

	**anterior**	**posterior**	**anterior**	**posterior**

CaP group (n = 4)	1/4	1/4	0/4	0/4
	4.3 ± 8.7% *	4.5 ± 9.0%	0%	0%
Control (n = 4)	4/4	3/4	0/4	2/4
	42.4 ± 20.4%	13.0 ± 13.4%	0%	14.4 ± 18.7%
				

AV: average, SD: standard deviation

* *p *< 0.05 compared with control values.

**Figure 7:**
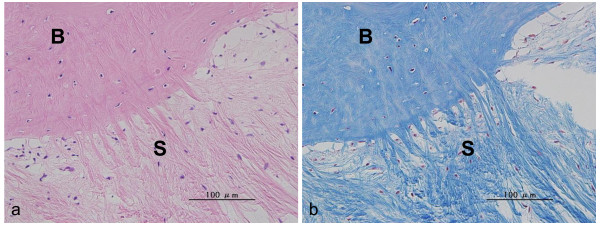
**Histological sections of control group**. This area is the anterior of the aperture site in the femur. (a) H-E staining (x 400), (b) MT staining (x 400), T = tendon graft, S = Sharpey's-like fiber, Sharpey's-like fibers penetrating the bone perpendicular to the direction of the lamellae were observed.

In the control group, Sharpey's fibers were dense and penetrated the bone perpendicular to the direction of the lamellae (Figure 7). Sharpey's fibers observed at the anterior bone tunnel were long. On the other hand, those at the posterior bone tunnel were short and dense. At the aperture site, cartilage layers between the tendon and the bone were observed at the anterior surface of the tibial bone tunnel in 3 specimens, at the posterior surface of the tibial bone tunnel in 1 specimen, at the anterior surfaces of the femoral bone tunnel in none of the specimens, and at the posterior surface of the femoral bone tunnel in 1 specimen.

**Figure 8:**
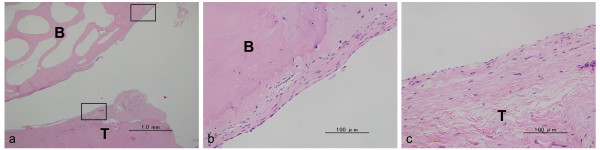
**Histological sections stained with H-E for the control group**. This area is the anterior of the aperture site in the femur. T = tendon graft, B = bone, (a) Low-magnification image (× 40). A gap area at the joint aperture site was observed. (b) and (c) are magnified views of the boxed part in (a) (× 400). A synovial tissue cover was observed on the tendon and bone tunnel surface.

The gap area in the femoral side in the control group was more observed than that in the CaP group (*p *= 0.0178). At the gap area, a synovial tissue cover was observed on the tendon and bone tunnel surface (Figure 8). The gap area between the tendon and the bone was observed at the anterior surface of the tibial bone tunnel in none of the specimens, at the posterior surface of the tibial bone tunnel in 2 specimens, at the anterior surfaces of the femoral bone tunnel in all 4 specimens, and at the posterior surface of the femoral bone tunnel in 3 specimens in the control group. The gap area rates were 0% in the anterior tibial bone tunnel, 14.4 ± 18.7% in the posterior tibial bone tunnel, 42.4 ± 20.4% in the anterior femoral bone tunnel, and 13.0 ± 13.4% in the posterior femoral bone tunnel.

In the CaP group, the gap area at the interface was small at the anterior surface of the tibial bone tunnel in none of the specimens, at the posterior surface of the tibial bone tunnel in none of the specimens, at the anterior surfaces of the femoral bone tunnel in 1 specimen, and at the posterior surface of the femoral bone tunnel in 1 specimen. The gap area rates were 0% in the anterior tibial bone tunnel, 0% in the posterior tibial bone tunnel, 4.3 ± 8.7% in the anterior femoral bone tunnel, and 4.5 ± 9.0% in the posterior femoral bone tunnel. The gap area rate in the anterior femoral bone tunnel in the CaP group was significantly smaller than that in the control group (*p *= 0.0069).

**Figure 9:**
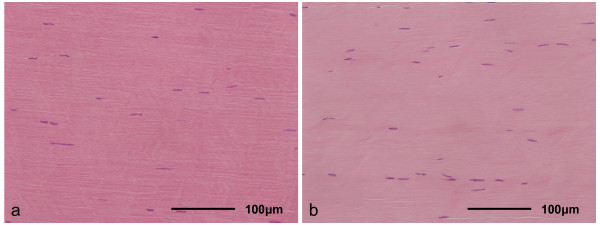
**Histological sections stained with H-E for the CaP group (a) and control group (b) (x 400)**. This area is the intra-articular portion. The microstructure of the tendon graft showed a uniform highly oriented collagenous matrix with interspersed spindle cells.

Two years after ACL reconstruction, the LTMIs of the tendon graft were similar in the CaP and control groups, in terms of the uniform linear collagen orientation and the spindle-shaped nuclear morphology of the graft (Figure 9). The LTMIs were 19.3 ± 3.9 for the CaP group and 18.5 ± 1.7 for the control group. There was no significant difference between the CaP and control groups (*p *= 0.7398).

## Discussion

We found direct insertion-like formation at the interface in the CaP group 2 years after ACL reconstruction. However, the structure was different from a normal insertion structure because it lacked tidemarks. The cartilage layer in the femoral and tibial bone tunnels at the anterior and posterior of the aperture site was more prominent between the tendon graft and the bone in the CaP group than in the control group. Mechanical load has significant effects on the formation, degradation, regeneration, and tissue composition of tendons in vitro and in vivo [21-25]. Yamakado et al. [26] demonstrated the regeneration of tendon-bone junctions at the entrance of bone tunnels after extensor tendons were grafted extra-articularly. They suggested that tension and/or compression enhances the healing of tendon-bone junctions and chondroid formation. In the CaP method, scarless direct bonding, which is due to the fact that the bonelike microstructure contains low-crystalline apatite and type I collagen, and reduction of inflammation are achieved in the early postoperative phase [13-15]. Then, the interface becomes a cartilaginous insertion 6 months after ACL reconstruction [16]. Moreover, the in situ force in the tendon graft is greater in the CaP method than in the conventional method 1 year after ACL reconstruction [17]. The interface with a CaP-hybridized tendon graft after direct bonding can react to tension and/or compression. The mechanical environment (tensile and/or compressive force) in this direct bonding probably further may promote the differentiation of the interface zone to a cartilage layer. Therefore, cartilaginous direct insertion-like formation can be realized 2 years after ACL reconstruction in the CaP group. However, we were unable to regenerate tidemarks, because the tendon-bone alignment in the reconstructed knee differs from that in the knee with normal ACL insertion. ACL insertion consists of four distinguishable tissue layers in transition, that is, ligaments, fibrocartilage, mineralized fibrocartilage, and bone [27,28]. The fibrocartilage and mineralized cartilage function as a stress or shock absorber by reducing the stiffness gradient between the ligament and the bone [29,30]. Reconstructing the highly specialized structure of cartilaginous insertion is necessary to reduce the transverse contraction of the ligament during tensile loading, which therefore acts as a stretching brake [29]. Joint aperture site healing with two distinguishable cartilaginous insertion layers (direct insertion-like formation) in the CaP group can neutralize graft motion within the tunnel. On the other hand, in the control group, mainly fibrous tissue intervention was observed at the interface between the tendon graft and the bone tunnel. A prolonged instability of the interface formed using an untreated tendon graft in the early postoperative period could lead to fibrous insertion formation owing to micromotion and inflammation at the interface for a long time. Shear stress at the interface associated with the bungee effect (longitudinal micromotion) [31], rotational micromotion, and transverse micromotion (windshield wiper effect) promotes fibrous insertion formation (Sharpey's fibers) in the control group, as similarly previously reported [3,13].

Bone tunnel enlargement in the CaP group was lesser in both the femoral and tibial bone tunnels than that in the control group. The possible etiologies for tunnel widening include synovial fluid cytokines, graft-tunnel micromotion, and inflammatory mediators. Synovial fluid influx into a bone tunnel may also affect healing. Berg et al. [32] studied the healing of empty bone tunnels in rabbit knees and found rapid bone formation at the extra-articular exit of a femoral tunnel, whereas bone formation was delayed at the intra-articular exit of the tunnel. They postulated that synovial fluid cytokines delay the healing of the intra-articular exit of the tunnel. Moreover, enlargement of the articular end of the bone tunnels is a common problem in ACL reconstruction [33], because the graft-tunnel motion can be greater at the tunnel aperture site than at the extra-articular end of the tunnel for grafts fixed by suspensory fixation. In the control group, a marked tunnel enlargement was observed by CT, and a histologically large gap area with synovial tissue covering the tendon graft and bone tunnel surface was also observed. Therefore, the continued micromotion at the interface can lead to bone tunnel enlargement not only on the femoral side but also on the tibial side when using untreated tendon grafts in our animal experiment 2 years after the operation. Moreover, bone tunnel enlargement on the femoral side 6 months after ACL reconstruction [9] could increase the gap area at the interface on the femoral side 2 years after the operation, because of micromotion and joint fluid. Synovial fluid pumping with cytokines caused by graft motion in the bone tunnel can promote synovial membrane formation on the tendon graft and bone tunnel surface. These phenomena may account for the loosening at the interface and knee instability in the control group. On the other hand, in the CaP group, the cartilage layer in the interface on the joint aperture site side can prevent the influx of synovial fluid and absorb the graft-tunnel motion. Therefore, bone tunnel enlargement was lesser and the gap area was smaller in the CaP group than in the control group.

The enhanced tendon-bone healing in the CaP group did not result in the maturation of the tendon graft. We considered that firm anchoring would be important for the maturation of the tendon graft. The midsubstance of the graft prepared using the CaP-hybridization method may promote the recovery of mechanical strength owing to its effective load transfer through the tibia-graft-femur construct after anchoring 1 year after ACL reconstruction in goats [10]. However, the maturation of the tendon graft in both groups was similar 2 years after ACL reconstruction. It may be difficult in terms of long-term results in animals to set appropriate mechanical conditions for ACL reconstruction by joint geometry.

Regarding the limitation of this study, since we did not perform mechanical analysis 2 years after ACL reconstruction, the mechanical strength in both groups is unclear in this study. Further study to clarify the mechanical properties of the tendon graft and to clarify the effect of mechanical stress on graft maturation is required. Examination of non-significant results of this study may be necessary to check for possible false negatives due to the small number of samples. If we use a larger number of specimens, a clearer significant difference may have appeared.

The CaP-hybridized tendon graft enhanced the tendon-bone healing 2 years after ACL reconstruction in goats. The CaP-hybridized tendon graft can reduce the bone tunnel enlargement and the gap area associated with the direct insertion-like formation in the interface at the joint aperture site.

## Competing interests

The authors declare that they have no competing interests.

## Authors' contributions

MS conceived of the study, and participated in its design and coordination. HM participated in the sequence alignment and drafted the manuscript. HM carried out the CT and histological analysis. All authors read and approved the final manuscript.

## References

[B1] Biau DJ Katsahian S Kartus J Harilainen A Feller JA Sajovic M Ejerhed L Zaffagnini S Röpke M Nizard R Patellar tendon versus hamstring tendon autografts for reconstructing the anterior cruciate ligament: a meta-analysis based on individual patient data Am J Sports Med 2009 37 2470 2478 10.1177/0363546509333006 19709991

[B2] Taylor DC DeBerardino TM Nelson BJ Duffey M Tenuta J Stoneman PD Sturdivant RX Mountcastle S Patellar tendon versus hamstring tendon autografts for anterior cruciate ligament reconstruction: a randomized controlled trial using similar femoral and tibial fixation methods Am J Sports Med 2009 37 1946 1957 10.1177/0363546509339577 19684298

[B3] Grana WA Egle DM Mahnken R Goodhart CW An analysis of autograft fixation after anterior cruciate ligament reconstruction in a rabbit model Am J Sports Med 1994 22 344 351 10.1177/036354659402200309 8037275

[B4] Nebelung W Becker R Urbach D Röpke M Roessner A Histological findings of tendon-bone healing following anterior cruciate ligament reconstruction with hamstring grafts Arch Orthop Trauma Surg 2003 123 158 163 10.1007/s00402-002-0463-y 12734713

[B5] Chen CH Chen WJ Shih CH Yang CY Liu SJ Lin PY Enveloping the tendon graft with periosteum to enhance tendon-bone healing in a bone tunnel: a biomechanical and histologic study in rabbits Arthroscopy 2003 19 290 296 10.1053/jars.2003.50014 12627154

[B6] Hashimoto Y Yoshida G Toyoda H Takaoka K Generation of tendon-to-bone interface ''enthesis'' with use of recombinant BMP-2 in a rabbit model J Orthop Res 2007 25 1415 1424 10.1002/jor.20447 17557323

[B7] Rodeo SA Suzuki K Deng XH Wozney J Warren RF Use of recombinant human bone morphogenetic protein-2 to enhance tendon healing in a bone tunnel Am J Sports Med 1999 27 476 488 10.1177/03635465990270041201 10424218

[B8] Huangfu X Zhao J Tendon-bone healing enhancement using injectable tricalcium phosphate in a dog anterior cruciate ligament reconstruction model Arthroscopy 2007 23 455 462 10.1016/j.arthro.2006.12.031 17478274

[B9] Sasaki K Kuroda R Ishida K Kubo S Matsumoto T Mifune Y Kinoshita K Tei K Akisue T Tabata Y Kurosaka M Enhancement of tendon-bone osteointegration of anterior cruciate ligament grafts using granulocyte colony-stimulating factor Am J Sports Med 2008 36 1519 1527 10.1177/0363546508316282 18413678

[B10] Martinek V Latterman C Usas A Abramowitch S Woo SL Fu FH Huard J Enhancement of tendon-bone integration of anterior cruciate ligament grafts with bone morphogenetic protein-2 gene transfer: a histological and biomechanical study J Bone Joint Surg Am 2002 84 1123 1131 10.2106/00004623-200207000-00005 12107310

[B11] Lattermann C Zelle BA Whalen JD Baltzer AW Robbins PD Niyibizi C Evans CH Fu FH Gene transfer to the tendon-bone insertion site Knee Surg Sports Traum 2004 12 510 515 10.1007/s00167-003-0482-4 15014945

[B12] Taguchi T Kishida A Akashi M Apatite formation on/in hydrogel matrices using an alternate soaking process: II. Effect of swelling ratios of poly(vinyl alcohol) hydrogel matrices on apatite formation J Biomater Sci Polym Ed 1999 10 331 339 10.1163/156856299x00397 10189101

[B13] Mutsuzaki H Sakane M Nakajima H Ito A Hattori S Miyanaga Y Ochiai N Tanaka J Calcium-phosphate-hybridized tendon directly promotes regeneration of tendon-bone insertion J Biomed Mater Res 2004 70A 319 327 10.1002/jbm.a.30084 15227677

[B14] Mutsuzaki H Sakane M Ito A Nakajima H Hattori S Miyanaga Y Tanaka J Ochiai N The interaction between osteoclast-like cells and osteoblasts mediated by nanophase calcium phosphate-hybridized tendons Biomaterials 2005 26 1027 1034 10.1016/j.biomaterials.2004.03.039 15369691

[B15] Mutsuzaki H Sakane M Hattori S Kobayashi H Ochiai N Firm anchoring between calcium phosphate-hybridized tendon and bone for anterior cruciate ligament reconstruction in a goat model Biomed Mater 2009 4 045013 10.1088/1748-6041/4/4/045013 19667461

[B16] Mutsuzaki H Sakane M Nakajima H Ochiai N Calcium phosphate-hybridized tendon graft to reduce bone tunnel enlargement after ACL reconstruction in goats Knee 2011 in press 10.1016/j.knee.2011.03.008 21514829

[B17] Mutsuzaki H Sakane M Fujie H Hattori S Kobayashi H Ochiai N Effect of calcium phosphate-hybridized tendon graft on biomechanical behavior in anterior cruciate ligament reconstruction in a goat model: novel technique for improving tendon-bone healing Am J Sports Med 2011 39 1059 1066 10.1177/0363546510390427 21220545

[B18] Jackson DW Grood ES Goldstein JD Rosen MA Kurzweil PR Cummings JF Simon TM A comparison of patellar tendon autograft and allograft used for anterior cruciate ligament reconstruction in the goat model Am J Sports Med 1993 21 176 185 10.1177/036354659302100203 8465909

[B19] Ng GY Oakes BW Deacon OW McLean ID Lampard D Biomechanics of patellar tendon autograft for reconstruction of anterior cruciate ligament in the goat: Three-year study J Orthop Res 1995 13 602 608 10.1002/jor.1100130416 7674076

[B20] Murray MM Spindler KP Ballard P Welch TP Zurakowski D Nanney LB Enhanced histologic repair in a central wound in the anterior cruciate ligament with a collagen-platelet-rich plasma scaffold J Orthop Res 2007 25 1007 1017 10.1002/jor.20367 17415785

[B21] Slack C Flint MH Thompson BM The effect of tensional load on isolated embryonic chick tendons in organ culture Connect Tissue Res 1984 12 229 247 10.3109/03008208409013685 6478823

[B22] Hannafin JA Arnoczky SP Hoonjan A Torzilli PA Effect of stress deprivation and cyclic tensile loading on the material and morphologic properties of canine flexor digitorum profundus tendon: an in vitro study J Orthop Res 1995 13 907 914 10.1002/jor.1100130615 8544028

[B23] Nabeshima Y Grood ES Sakurai A Herman JH Uniaxial tension inhibits tendon collagen degradation by collagenase in vitro J Orthop Res 1996 14 123 130 10.1002/jor.1100140120 8618154

[B24] Evanko SP Vogel KG Ploteoglycan synthesis in fetal tendon is differentially regulated by cyclic compression in vitro Arch Biochem Biophys 1993 307 153 164 10.1006/abbi.1993.1574 7694546

[B25] Giori NJ Beaupre GSB Carter DR Cellular shape and pressure may mediate mechanical control of tissue composition in tendons J Orthop Res 1993 11 581 591 10.1002/jor.1100110413 8340830

[B26] Yamakado K Kitaoka K Yamada H Hashiba K Nakamura R Tomita K The influence of mechanical stress on graft healing in a bone tunnel Arthroscopy 2002 18 1 82 90 10.1053/jars.2002.25966 11774147

[B27] Benjamin M Evans EJ Copp L The histology of tendon attachments to bone in man J Anat 1986 149 89 100 PMC1261636 3693113

[B28] Cooper RR Misol S Tendon and ligament insertion: a light and electron microscopic study J Bone Joint Surg Am 1970 52 1 20 4189231

[B29] Knese K-H Biermann H Osteogenesis in tendon and ligament insertions in the area of the original chondral apophyses Z Zellforsch Mikrosk Anat 1958 49 142 187 13625935

[B30] Schneider H Zur Struktur der Sehnenansatzzonen Z Anat 1956 119 431 456 13353255

[B31] Höher J Livesay GA Ma CB Hamstring graft motion in the femoral bone tunnel when using titanium button/polyester tape fixation Knee Surg Sports Traum 1999 7 215 219 10.1007/s001670050151 10462210

[B32] Berg EE Pollard ME Kang Q Intraarticular bone tunnel healing Arthroscopy 2001 17 189 195 10.1053/jars.2001.20958 11172249

[B33] Rodeo SA Kawamura S Ma CB Ma CB Deng XH Sussman PS Hays P Ying L The effect of osteoclast activity on tendon-to-bone healing: an experimental study in rabbits J Bone Joint Surg Am 2007 89 2250 2259 10.2106/JBJS.F.00409 17908903

